# A qualitative dyadic approach to explore the experiences and perceived impact of COVID-19 restrictions among adolescents and their parents

**DOI:** 10.1080/21642850.2023.2173601

**Published:** 2023-02-04

**Authors:** Pooja Saini, Anna Hunt, Joanna Kirkby, Jennifer Chopra, Emma Ashworth

**Affiliations:** School of Psychology, Faculty of Health, Liverpool John Moores University, Liverpool, UK

**Keywords:** COVID-19, parent–child dyad, homeschooling, qualitative, working from home

## Abstract

**Background:**

While evidence exists for the negative and positive effects of the COVID-19 pandemic and associated lockdown on the mental health and well-being of adolescents and parents separately, the potential impact of lockdown, and the effective coping strategies that have been used have so far, by both children and their parents still needs to be explored.

**Method:**

A dyadic approach was used to explore the perceived impact of COVID-19 restrictions among early adolescents and their parents in Northwest England. Nine parents (8 female and 1 male) and their 10 children (6 boys and 4 girls) aged 11–13, were recruited from 4 secondary schools to be interviewed. Remote interviews took place between October and December 2020 for the adolescents and between March and May 2021 for their parents. Inductive thematic analysis was used.

**Results:**

Five inter-related themes were identified: (1) overcoming barriers for learning at home; (2) juggling a work–life balance; (3) loss of experiences; (4) caring for other family members; and (5) adopting new self-care and coping strategies during the pandemic.

**Conclusion:**

Themes identified will help to inform policy and practice for supporting adolescents and parents in the future, including the promotion of positive coping strategies and the provision of resources for adolescents, schools and families.

## Introduction

Worldwide restrictions to prevent infection during the COVID-19 pandemic have included isolation, social distancing, and school closures (Gov.UK, 2021). Whilst these measures are implemented to reduce the spread of infection and prevent loss of life (British Medical Association, 2020), such measures can have a negative impact, particularly on child health and well-being (Irwin et al., 2022; Rajmil et al., 2021), and mental health (Elharake et al., 2022; Liang et al., 2020). In the UK, repeated school closures during national lockdowns (23rd March–4th July 2020 and 5th November 2020–8th March 2021), alongside community-wide Governmental advice such as staying at home, reducing social contact, and closure of leisure and retail businesses, have likely resulted in reduced face-to-face contact with peers and family members and changes to routines that are difficult to adjust to (Lee, 2020).

Lockdown measures have substantial societal effects, including a significant impact on parents with younger children and adolescents, with significant changes to daily routines and family functioning (Solmi et al., 2022). School closures shifted a large part of the responsibility for children’s education to parents within the home environment; this all but obliterated the notion of a healthy work–life balance, where competing time demands and the sudden precariousness of their economic position meant that working parents had to endure financial distress and a deterioration of their well-being, especially their mental health (Cheng et al., 2021; Yerkes et al., 2020). Evidence points to the importance of family contexts in relation to the impact of lockdown, with groups such as key workers, those with histories of mental or physical health conditions, those from disadvantaged communities, Black, Asian and Minority Ethnic (BAME) groups, and those affected by violence or abuse being more likely to be negatively affected by lockdown (Millar et al., 2020). The way in which parents have been impacted by the COVID-19 pandemic also reflects a complex gendered reality (Cheng et al., 2021; Kerr et al., 2021; Sevilla & Smith, 2020; Yerkes et al., 2020) and discrepancy between working parents who experience significantly higher levels of financial distress relative to working counterparts without children (Cheng et al., 2021). During lockdowns, mothers have reported more adjustments in the times at which they work and experienced more work pressure in comparison to before the lockdown compared to fathers (Cheng et al., 2021; Kerr et al., 2021; Sevilla & Smith, 2020; Yerkes et al., 2020). Moreover, mothers continue to do more childcare, including emotional support and household work than fathers, although some fathers report taking on greater shares of childcare and housework during the lockdown in comparison to before (Yerkes et al., 2020). Mothers also report a larger decline in leisure time than fathers. Employers are less sensitive to the additional hours of childcare done by women compared to men, leaving many women juggling work and (a lot more) childcare, with likely adverse effects on their mental health and future careers (Sevilla & Smith, 2020). However, there has been some evidence of increased positive emotions in parenting as well, including feelings of closeness to children and gratitude (Kerr et al., 2021).

Although many parents will show resilience in the face of the challenges associated with COVID-19, for many others, the prolonged lockdown and lack of support will likely exacerbate existing vulnerabilities and contribute to the onset of new stress-related disorders (Horesh & Brown, 2020). For example, services for neglected children and children in ‘at-risk’ families (e.g. daily educational centres) were suspended during the lockdown, with only a few continuing to provide remote support, with significant challenges.

Furthermore, there is sparse research exploring the specific COVID-19 related factors that may boost adolescents’ well-being during the pandemic, such as family shielding (protecting family members defined on medical grounds as extremely clinically vulnerable to COVID-19) or keyworker status (people working within health and social care, education and childcare, public safety and national security, transport, utilities and communication, food and other necessary goods, financial services, key national and local government), and individuals’ understanding of and level of worry regarding COVID-19 (Ashworth, Putwain, et al., 2022). Recent research indicates that the ‘ordinary magic’ of supportive relationships for adolescents and positive experiences appear to be some of the key factors needed to maintain adolescents’ mental health and well-being, and to help them overcome difficulties posed by the COVID-19 pandemic (Ashworth, Putwain, et al., 2022). However, more research is needed on both parents and their children’s experiences of COVID-19 in England to understand the impact on families.

Recent studies about parents’ home-schooling experiences during the COVID-19 period found that parents reported having difficulties (Nayir & Sari, 2021; Thorell et al., 2022) and high levels of psychological distress and social impairment (Calear et al., 2022). They also reported that home-schooling put too high demands on children (Thorell et al., 2022). These included their children not being able to concentrate or fully participate in home-schooling, completing the work required within a reasonable time, parents needing to manage multiple children’s lessons at the same time, and the lack of internet or poor connection. Parent’s report a variation in how many hours (1–4 h) children worked for during their home lessons and how they received information about their course schedules from their teachers. Research has shown that parents felt communication between teachers and children to be important (Nayir & Sari, 2021) and that in some cases it was insufficient or very limited (Thorell et al., 2022).

Digital exclusion has been a significant issue for families during the pandemic, with school closures highlighting the gaps in education caused by low income, with children unable to access or engage in learning due to inadequate resources (Child Poverty Action Group, 2020). Forty per cent of low-income families indicated that they were missing at least one essential resource (Child Poverty Action Group, 2021). For parents who had computer devices with internet access at home, children may still not have achieved optimum online learning, particularly as some parents needed to work from home, making it impracticable to devote quality time to ensuring adequate learning was being achieved (Briggs, 2020; Parczewska, 2021). Four out of 10 parents reported providing a supervisory role for children completing schoolwork, some tried to help by explaining difficult topics and most provided their children with a designated learning space (Briggs, 2020; Parczewska, 2021). Some parents scheduled a structured schooling day with breaks factored in, others did not ensure that their children did physical activity during the day, some admitted feeling frustrated and reported increased conflict, yelling, and discipline, and others the feeling of hopelessness related to the lack of ways or abilities to effectively motivate the child to learn (Kerr et al., 2021; Parczewska, 2021). However, many parents reported no changes in parenting behaviours, and some noted increased comfort and praise (Solmi et al., 2022).

Parents shared worry about the negative impact on their children’s education and that they found the home-schooling situations very difficult (Nayir & Sari, 2021; Parczewska, 2021; Thorell et al., 2022). Additionally, a large proportion of parents (50% or above) reported that they and their child felt more isolated and that the child used digital media more often for things besides schoolwork (Thorell et al., 2022). Previous findings also highlighted the impact of severe restrictions on vulnerable populations’ well-being and mental health outcomes, including children, adolescents, and those with autism spectrum disorder (ASD) (O’Sullivan et al., 2021). Overall, research has recognised that the mental health impacts of home-schooling are high and may increase with its duration and frequency (Calear et al., 2022). Positively, some parents gained more insight into their children’s learning and felt they had the opportunity to contribute, have good discussions in various subjects and were now in a better position to help their children with schoolwork (Bubb & Jones, 2020; Thorell et al., 2022). Others reported being able to give their children more comfort and praise when home-schooling (Kerr et al., 2021) and that bullying was reduced (Thorell et al., 2022).

While evidence exists for the negative and positive effects of the COVID-19 pandemic and associated lockdown on the mental health and well-being of adolescents (Ashworth, Hunt, et al., 2022; O’Sullivan et al., 2021) and parents (Nayir & Sari, 2021; Parczewska, 2021; Thorell et al., 2022) separately, the potential impact of lockdown, and the effective coping strategies that have been used have so far, by both children and their parents still needs to be explored. Additionally, the evidence base has mostly involved quantitative research and has been conducted outside of the UK. While this is valuable in terms of identifying the scale of the disruption the pandemic has caused, it does not allow for subjective, individual experiences, or acknowledge the heterogeneity in the early adolescent population and their parents (Dvorsky et al., 2021). Indeed, the voice of adolescents and their parents is a valuable resource both during and after the COVID-19 pandemic (Branquinho et al., 2020).

The study aims to use a dyadic approach (Manning & Kunkel, 2015) to explore the experiences and perceived impact of COVID-19 among early adolescents and their parents in Northwest England, and the self-care and coping strategies that helped them. These insights may help to inform policy and practice when supporting adolescents and their parent’s post-pandemic, and during any future similar events. In line with the aforementioned aims, research questions are as follows:

1. What are early adolescents’ and their parents’ experiences of lockdowns and return to formal schooling during the COVID-19 pandemic?
2. What self-care and coping strategies do early adolescents and their parents describe using during the COVID-19 pandemic to support their mental health and well-being?

## Methods

### Design

A dyadic approach was used to explore the perceived impact of COVID-19 restrictions among early adolescents and their parents in Northwest England. Nine parents (8 female and 1 male) and their 10 children (6 boys and 4 girls) aged 11–13, were recruited from 4 secondary schools to be interviewed. Remote interviews took place between October and December 2020 for the adolescents and between March and May 2021 for their parents. The current study used qualitative interview data collected remotely as part of the Adolescents’ Lockdown-Induced Coping Experiences (ALICE) study (Ashworth, Hunt, et al., 2022).

### Participants and recruitment

All adolescent participants attended secondary schools in Northwest England. Two schools were coeducational and two were single sex (one boys’ and one girls’). The single sex schools were both academically selective, and one school was fee paying. Across the four schools, 65 adolescents consented to be contacted for a qualitative interview. The parents/carers of all potential participants were sent an email containing an information sheet and a parent consent form. Of those, 17 parents expressed interest in the interview and 14 consented for their child to take part. In total, pupils from four schools took part in the interviews, with five of the adolescents attending the first school, and the remaining students attending the three others.

The mean age for child participants was 12 years (range 11–13 years) with 6 boys taking part and 4 girls. Four of the interviewees were from BAME groups, and the remainder were White British. Eight of the parents who took part in the interview were female, and one was male. One interviewee was from a BAME group, and the remaining eight were White British.

### Procedures

Schools sent information sheets to the parents/carers of all pupils in these year groups, along with a link to an online survey, consisting of a suite of measures exploring adolescents’ mental health and well-being during the COVID-19 pandemic, and their experiences of lockdown. Two hundred and ninety-four adolescents participated in the survey. At the end of the survey, adolescents were asked to provide their parents’ contact details if they would like to opt-in to a follow-up online one-to-one qualitative interview. The parents of these adolescents were then contacted, and their opt-in consent sought. Corresponding parent online interviews took place as pupils were set to return to face-to-face schooling. Nine parents of 10 children who took part in the original ALICE interviews participated. Two of the adolescents were twins and one parent was interviewed for them. Parents were contacted by the research team and provided with a report of the original ALICE study findings, along with an invite to take part in a follow-up one-to-one qualitative interview about their experience of home-schooling during the Covid-19 pandemic.

### Data collection

Data was collected using one-to-one semi-structured interviews. Two separate tailored semi-structured schedules were developed for the interviews, one for parents and one for adolescents. The schedules were designed to act as a guide to ensure specific topics were addressed, whilst also allowing for unexpected responses (Galletta, 2013). Adolescents’ interviews lasted on average 26 min (16–41 min). Parent interviews lasted on average 48 min (41 min to 1 h 15 min).

Broad topics covered in the interview schedules included well-being during the pandemic, coping strategies, and resilience processes that promoted well-being such as relationships with family and friends, home life, experiences of and feelings towards lockdown and COVID-19, working-from-home and changes to schooling. An example question was ‘I wondered if you could tell me a bit about what home-schooling has looked like for you during lockdown? What did you think of this?’. Prompts and probes were utilised where required to reassure participants to elaborate on their answers and clarify unclear responses.

Due to COVID-19 restrictions, the interviews took place online via Microsoft Teams. Fully informed opt-in consent was taken prior to the interview beginning. To account for ethical concerns when interviewing adolescents, care was taken to reduce power imbalances and to ensure participation was fully informed and voluntary. For example, prior to starting the audio recording, the researcher introduced themselves to the adolescents and engaged in conversation to build rapport (Arksey & Knight, 1999; Eder & Fingerson, 2003). For both the adolescents and parent interviews the researcher explained the study, reiterated that the study was voluntary and that they could stop at any time, and offered an opportunity to ask questions. Adolescents were offered the option to choose their own pseudonym to increase feelings of control and ownership of their data (Allen & Wiles, 2016). Additionally, parents were given a participant ID number by the Researcher which was linked with their child’s pseudonym.

### Analytic strategy

Transcripts were analysed by five authors (PS, AH, JK, EA, JC). The audio recordings were transcribed verbatim using the Otter programme (www.otter.ai), reviewed by a researcher for accuracy and analysis took place in NVivo 11 (https://www.qsrinternational.com/nvivo/). Braun and Clarke’s (2006, 2019) reflexive thematic analysis was used to analyse the data. We adopted an inductive approach, focusing on what was personally meaningful to participants. This approach aligned with our focus on developing rich experiential themes that could capture latent features of the data (rather than purely semantic) and thus offer nuanced insights into participants’ subjective experiences.

Authors (PS, AH, JK) began analysis on paired dyad transcripts first, familiarising themselves with the dataset and generating initial codes. Dyadic analysis was used as the core stage of analysis. Dyadic analysis in qualitative research allows for researchers to better understand and identify overlaps and contrasts between the pair interviewed, particularly if they have been interviewed separately. This, in turn, enables researchers to see beyond the individual perspectives and into the perceptions of their experiences as a dyad (Manning & Kunkel, 2015). After approximately 25% of the transcripts had been coded, these codes were explored to develop an initial set of themes. All authors analysed the final 75% of the transcripts, reviewing the themes and developing as appropriate. Once all data had been analysed, authors (PS, AH, JK, EA, JC) engaged in reflexive discussion to explore the decisions made and examine where further consideration may be valuable; the thematic structure was then revisited and developed in line with this discussion. The approach taken was also informed by quality and rigour principles and guidance, particularly that outlined by Yardley (2000) of sensitivity to the research context, commitment and rigour, transparency and coherence, and impact and importance.

### Ethics statement

The ALICE study received ethical approval from Liverpool John Moores University Research Ethics Committee (Ref: 21/PSY/006 and 20/NSP/037).

## Findings

Nineteen interviews were conducted with 10 adolescents (4 girls and 6 boys) and 9 of their parents (8 mothers and 1 father). Table 1 shows the characteristics, family relationships, parent work situation and key discussions from the one-to-one interviews. Five out of nine parents identified as a Key Worker (e.g. Doctor/Nurse/Teacher, etc.). Only one adolescent lived at home with just their parents, whilst the remaining lived with siblings too. Two adolescents had grandparents move into their house during the first lockdown.

**Table 1.** Main discussion points of the parent–child dyad pairings.

Parent–child dyad	Relationship	One or more parent working from home (during at least one lockdown)	Main discussion points
Dyad 1	Mother/Twin sons	Yes	Positive experience (time together as family); Feel lucky; Disappointed SATS didn’t go ahead; Transition into Secondary School not typical.
Dyad 2	Mother/Son	No	Younger sibling had a different experience; Technology issues; Lack of routine; Both parent and child isolated from friends; Feel lucky; Positive experience (time together as family).
Dyad 3	Mother/Son	Yes	Parent keeping child motivated; Lack of feedback from teachers; Technology/equipment issues; Positive experience (time together as family).
Dyad 4	Mother/Daughter	Yes	Positive experience (time together as family); Maintained friendships through technology; Harder to focus on schoolwork; Parent had to motivate younger sibling; Relaxed routine.
Dyad 5	Mother/Daughter	Yes	Enjoyed working at home; Enjoyed making own routine; Lack of feedback from teachers; Technology issues; Maintained friendships through technology.
Dyad 6	Father/Son	Yes	Grandma joined bubble; Enjoyed making own routine; Did schoolwork early in day to have free time later on; Lack of contact with friends; Positive experience (time together as family).
Dyad 7	Mother/Son	Yes	Challenging for parent; Parent helped with schoolwork in 1st lockdown; Overwhelming for child; Harder not having teacher there; Lack of social contact; Maintained friendships through technology.
Dyad 8	Mother/Daughter	Yes	Low motivation for home-schooling; Lack of feedback from teachers; Lack of routine; Maintained friendships through technology; Transition to Secondary School not typical; Difficult for parent to juggle home school and work/child care.
Dyad 9	Mother/Daughter	Yes	Change in home location between lockdowns; Family separated for period due to location change; Low motivation for home-schooling; Maintained friendships through technology; Positive experience (time together as family); Difficult for parent to juggle home school and work.

Following the thematic analysis process, five inter-related themes were conceptualised as reflecting the corpus of this material. The first theme illustrated respondents overcoming barriers for learning at home. The second theme discussed the parents juggling a work–life balance. The third theme related to the impact of COVID-19 on the loss of experiences, particularly for adolescents starting secondary school. The fourth theme highlights the impact of caring for other family members and how people communicated using online technology. The final theme explored both adolescents and their parents adopted new self-care and coping strategies during the pandemic.

### Theme 1: overcoming barriers for learning at home

During the first lockdown, adolescents gave mixed responses to working from home, but most were motivated to keep up with their work, although they reported finding it harder without having a teacher present;

Because I didn’t have like a teacher to tell me really what to do, it just felt a bit weird having to do it without orders. When it’s gone back to normal, it feels a lot, a lot more relaxed and stuff. (Dyad 7, Child)

Parents were motivated to keep their children’s learning going and they felt when schoolwork was provided this prevented boredom. Parents reported it being easier to manage home-schooling for children who were the same age or who were older and more independent, rather than multiple children across different year groups. Further challenges were evident with younger children who needed more motivation and encouragement from their parents more regularly or where their children were in different schools:

Initially it was hard in terms of the home learning as the children were at three different schools. So, we had lots of communication from the different schools … there was very little interaction with the teachers, which the youngest one in particular found really difficult. (Dyad 5, Parent)

Parents reported that they felt the first lockdown had a negative impact on their children’s learning as they were provided with less work, were lacking focus, and needed to make their own routine. Where work was provided, there were reports of the workload being too heavy, varied support from teachers alongside the work and there was also a lack of feedback on work, thus making it more difficult for parents and children to keep track of their progression;

It was really hard because, because the teachers they … some of them weren’t as supportive, I guess as others. So, I would submit some work and some teachers would get back straight away, and some of them would leave it until quite a while. I guess that had knocked my confidence a bit because I didn’t know if I was doing it right or wrong. (Dyad 5, Child)

Parents discussed their children becoming angry and frustrated with home-schooling and the difficulty of trying to encourage some of their children to complete their work;

He struggled with that [being followed up to complete schoolwork] and trying to get him to do any work, he would just get quite upset, quite angry. And I think I learned quite early on I thought, actually that’s not what is important at the minute he will catch up with the academic learning. Let’s just focus on keeping healthy and happy. (Dyad 9, Parent)

All interviewees reported differences in home-schooling from the first to the second lockdown. These included having more structured teaching during regular school hours with live lessons, heavier workloads, more feedback from teachers, and children needing less input from their parents;

For a fact I think virtual is much better than uploading things online, because when you upload things, you can’t really ask questions, you can’t really understand that much. And for like, online, it’s much more better because you can like ask questions, and you can raise your hand on like the computer and stuff. (Dyad 9, Child)

Overall parents reported these differences as positive but some continued to encounter difficulties during the second lockdown;

To summarise it, the whole home-schooling situation had been ramped up massively. You know, there’s Government directives now that kids are meant to be in school at home for the same amount of time that they would be in school and that massively affected us … the fact that there’s so much more intensity expected of the kids and the parents as well, even though there’s no pressure and you’re not a teacher, and all the rest of it, you know, really has made for a very, very difficult home situation for us. (Dyad 3, Parent)

Parents also worried about the amount of time children needed to be in front of a computer screen;

I think it’s very, very difficult to sort of like expect a kid who’s got a low concentration span to actually sit in front of a PC all day or for three hours because that’s certainly what it was, from nine till quarter past 12. (Dyad 7, Parent)

For some adolescents the increased workload in the second lockdown caused higher levels of anxiety, particularly where there was a lack of engagement with teachers and schoolwork was not being completed. Other adolescents worried about returning to school and being in trouble with their teachers not completing their work;

Probably just like the worry about, what my teachers are going to say when I get back to school … like probably just worrying about like what the teachers would say if, like, if I didn’t do some of my *work*, would they like shout at me when I got back and stuff, but they didn’t so it’s fine. (Dyad 4, Child)

### Theme 2: juggling a work–life balance

Nine out of 10 of the children interviewed had at least one parent working from home during lockdown. A reoccurring theme was that parents enjoyed working from home and having the ability to balance work and childcare;

So, I actually found the initial lockdown quite good as a family, just spending time together. Just sort of going for walks. (Dyad 6, Parent)

Overall, the interviewees enjoyed the flexibility of working from home and some children even enjoyed completing their schoolwork early and being free for other activities;

We can do the work within our own time sort of thing. So if you did want to say, oh, come on, let’s go out for a nice walk. We can just do that. So you’ve got that flexibility. (Dyad 4, Parent)

However, there were both positive and negative aspects to working from home. Some people were happy staying at home and reported being more productive and enjoying time away from the office. Others reported more challenging aspects to working from home, such as the lack of engagement with work colleagues, difficulty in learning new skills from home and maintaining contact with friends. There was also the need for balancing other caring responsibilities that they may have previously had family support for, and increased fatigue. For families working from home and home-schooling, there was a need to balance everyone’s requirements with regards to access to technology;

The technology has been the thing that’s been a bit challenging. [Child name] kept having to use my Kindle at the beginning. And that, again, we kind of worked around and sorted out. There’s been a lot of arguments about who’s using the printer and you know, having to share resources. (Dyad 9, Parent)

Lack of usual equipment and technical issues were reported to be problematic especially when adolescents were required to login for online lessons or upload schoolwork and sometimes this did not register and parents would be followed up by the school;

The computer we’re on now is a very old one, and it can overheat, and it did it in the middle of your interview, it will do things like that. And then for [child name 1], he’s got his own computer. So he’s not too bad. But for [child name 2] it’s hard then to try and get him back engaged. And I know with this lockdown, with all these live lessons and things, there was, I think, 12 live lessons a week that [child name 2] should have been attending. And my mum could see he was attending them I’d seen he was attending, so but for some reason, the way his computer was logging on to them, the computer wasn’t recognising it was him. So it would say he’s only attended one live lesson this week. So you felt like you were in a battle with school sort of saying, He’s obviously doing it? And some other teachers would reply and go no, we know, you know, he’s interacted or he’s answered the question or has been doing some work. But when you’re in work, and you’re getting the message from school to say he’s only attended one of the live lessons last week, it’s not very productive, either. (Dyad 2, Parent)

There were further challenges for all family members having access to Wifi and technology at the same time as parents needed to complete their work whilst children also needed to access their online resources or be part of live lessons. Some parents aired their frustrations with schools setting unreasonable rules for children having technical issues;

Do not put these stipulations on the kids that they can’t join after 10 minutes because half the time it’s connection issues. You know, I have sat there with him and we cannot get connected. We live in a house, it’s an old house, it’s got thick walls when everybody’s working using the Wifi, it’s really difficult sometimes to get on. We’ve got four people trying to get onto laptops all at the same time. (Dyad 3, Parent)

Five of the parents within the study were keyworkers but not all had to work away from home. Two participants reported their mothers needing to move in during lockdown to help look after children. Parents who were keyworkers felt guilty if they were furloughed to look after their children and their colleagues were working in risky environments or they worried about risking their families if they continued working in front line jobs whilst feeling guilty being away from home;

So, for me, I felt a lot of guilt, I think as a single parent anyway, you do feel guilt, but I felt guilt that my mum had to stay here. And then I had guilt of was I bringing anything back to the home because the three of them were safe in the bubble of the house. And you know, they would go in the garden and play and things but they weren’t mixing with other people. Whereas it was me that was going to work or it was me that was going to the shops. (Dyad 2, Parent)

### Theme 3: loss of experiences

Parents and some adolescents, reported the disappointment with the year 6 SAT [Standard Assessment Tests] exams being cancelled; however, some adolescents were relieved to not have to complete the exams;

My kids missed their SATs as well, which is such a big thing, isn’t it in the big rite of passage almost really. (Dyad 1, Parent)

Well, I was preparing for my SATs and stuff with my mocks. It’s just like your mocks were pointless, basically because you don’t actually get to do your SATs. So, it made me feel quite upset. (Dyad 1, Child)

The transition from primary to secondary school and the experience of starting secondary school was affected negatively by the social distancing rules. Many parents felt disappointed that their children had limited opportunities to make new friends and explore their new school in way they would have if the rules were not in place;

They didn’t have their transition days the way that they would have done … One thing that I would say is that I do feel as though they’ve been a little bit sheltered from the secondary school experience. Because they have gone in and they’ve gone into a class together, and they stayed in that class and the teachers come to them, it’s not what I would associate as a traditional sort of secondary school experience, when you get plunged in you find your way around big buildings. (Dyad 1, Parent)

Many adolescents reported having to stay in bubbles in school and not being able to interact with peers freely. Additionally, being sent to work from home again all of a sudden if there was a covid case within their bubble.

You’ve seen people who you’ve never met before, which was a little bit hard to like, on the first day it was like we’d all go into class and we had like two hours of form time. So two hours to get to know each other … I found it a little bit awkward cause we’d just gone in for two weeks, so we’d got used to the work and we’d settled in and then on last Friday in the morning, we were immediately sent home, so I was like oh no I was like what was happening and then the teachers told us that we’d have to go home, so that was hard because you’ve like, you’ve got ready for school and then you’ve just got to go immediately home. (Dyad 1, Child)

Both parents and adolescents brought up not being able to socialise with their friends as they did before and how this was one of the biggest adjustments during lockdown;

It was hard, obviously we have a good social life, and not having that social life anymore, and not seeing all your friends. That was the only hard thing about it [lockdown]. (Dyad 6, Parent)

Adolescents did not have the opportunities to be part of their usual activities such as sports clubs or choir at school and therefore participated in less exercise and were not able to see their friends regularly;

Yeah I do *netball*, and then I was gonna start doing rounders. And I also do running but obviously, we don’t do them now because it’s just like extra time in school and more like chance to spread it and stuff. So we don’t do the after school clubs anymore. (Dyad 4, Child)

It was a gradual change with activities like choir, when COVID first got into the UK it was stopped because of the obvious risks, then band started to stop, and then fencing stopped because we shared equipment and you have to wear masks, so it was a really gradual downhill slope. (Dyad 3, Child)

Interviewees brought up holidays being cancelled and how that impacted negatively on families as they missed out on significant activities that would have previously taken place;

We thought we were gonna go on holidays somewhere, but obviously we couldn’t book anything. (Q: And how did that make you feel?) Annoyed because going on holidays is the best bit of the year and we missed it. (Dyad 7, Child)

### Theme 4: caring for wider family members

The impact of covid for different family members varied across the sample of interviewees with regards to caring responsibilities. Some had to move in, some move out, others be stricter with social distancing and there was a sense of increased worry for each other’s well-being. Parents worried more about how the changes affected their children and equally children worried about their parents;

Grandma moved in with us over lockdown, and we didn’t see mum for most of it because she was working for longer hours … (Q: How this made you feel?) It made me feel a bit weird because I thought she might feel a bit lonely. (Dyad 2, Child)

Lockdown rules sometimes had negative impacts, particularly when needing to take care of vulnerable family members. Within some families, grandparents joined their bubbles to ensure they were not alone and isolated. However, this would impact on families where younger children had to stick to the rules more stringently and maybe socialise less with their friends;

Included in our bubble is my dad who’s 78 years old. So, in the beginning of lockdown, he was very much isolated in his house, and I would be the only person who would go there. Then when we were allowed to have bubbles, because he is clinically very vulnerable, we still had a period when I was still going there, even though he was allowed to come here. But since you know, just more recently, he’s had the vaccine now, but we have been absolute sticklers for keeping to the rules. (Dyad 3, Parent)

Adolescents reflected on how lockdown restricted their loved ones from caring for family members, especially those who died in hospital alone after being unwell. They also spoke empathetically about their friends who lost family members during lockdown;

So my dad used to go and see her but she became very ill. My dad was balancing work and he needed to go and see her every day, and me and my mum we used to spend a lot of time in hospital and things, but when COVID got really bad, we couldn’t … we needed to … my dad needed to go up and see her but she got put in hospital and she passed away and my dad was very upset and he doesn’t usually get upset, so it was very like sad watching him be like that. (Dyad 8, Child)

### Theme 5: adopting new self-care and coping strategies

Parents and adolescents spoke about seeing their friends less and what helped them to manage their well-being during lockdowns and home-schooling. Everyone reported how they enjoyed their time together at home as a family, and the positive outcome of more bonding within their family;

Well, I’ve got, like, to spend more time with like, my family, and with my sister and stuff. Because usually we just like, would say bye in the morning, then when we come back, we’d just go on our phones and stuff, and not really speak to each other but we’ve had, like, more time together and stuff so that’s good. (Dyad 4, Child)

I think I would be more distant with my friends because of this lockdown, usually I would like meet with them and go to their houses and everything. But because like their parents are worried about like, the Coronavirus and stuff, we can’t really meet with each other, but I’ve definitely gotten much more closer to my parents than I usually would be, which was nice. (Dyad 9, Child)

There were many practical ways interviewees improved their well-being through exercise, maintaining normal activities, playing board games together and by connecting with friends and family virtually. Concerning COVID-19, respondents reported the importance of following the guidelines and keeping safe. Regarding home-schooling, planning, routine and regular contact with teachers were vital for ensuring schoolwork was completed in a timely manner. Some advice respondents said they would give to others to stay happy during the lockdown period was being realistic about the situation, helping and supporting each other, putting mental health and happiness first, not comparing yourself to others, keeping entertained, following guidelines/keeping safe, enjoying time together, exercise/physical activities, playing together, virtual reality, talking, and taking a break from work;

I think keep yourself entertained, maybe try finding a few TV shows, maybe if you’re like my age, find yourself a few games that you can try and play. You’ve got online bookstores and stuff. You’ve got family that you can maybe play games with. (Dyad 6, Child)

I just think my advice would be enjoy the time with your family and your children, very much so, because we’ll probably never be in this situation ever again. (Dyad 8, Parent)

If I had to sum it up, it would be don’t compare yourself to others. And what you’re doing for your family is what you think is the best for your family and don’t compare yourself to other people because they lead totally different lives and what might be great for them, won’t necessarily be good for you. I think, you know, if I have to say a mantra I’d say make sure you’re present every day. (Dyad 3, Parent)

## Discussion

This study explored the experiences and perceived impact of COVID-19 among early adolescents (aged 11–13 years) and their parents in Northwest England, and the self-care coping strategies that helped them. Conducting dyadic analysis yielded interesting results by highlighting the dynamics of relationship processes in parents’ and adolescents. Dyadic summaries were created and allowed for a clearer understanding of parents’ and adolescents’ perceptions of their experiences at a dyadic and individual level, what coping mechanisms they put in place to manage their experiences together and what challenges they faced.

Parents reported that the first lockdown had a negative impact on their child’s learning, regardless of the amount of work provided by the school. Some parents believed that there was not enough work provided, whereas some parents reported too much work was given. Contact with a teacher appeared to be particularly important for the adolescents interviewed as they found it harder to learn from home without a teacher being physically present. Parents comments mirrored this, as they reported that their adolescents did not always receive adequate support and feedback from teachers and that regular contact with a teacher was essential to the maintenance of education. This finding corresponds with research by Thorell et al. (2022), who found that adolescents in the UK had minimal contact or no contact at all with their teachers during periods of remote learning. The importance of having face-to-face contact with a teacher for adolescents’ education has also been highlighted by Ashworth, Hunt, et al. (2022). As such, adequate teacher support and feedback must be maintained during any future periods of remote learning. However, evidence suggests that teachers, who are already a group who experience high levels of burnout and stress (Long & Danechi, 2021), struggled to cope with the additional responsibility placed on them during lockdowns, and that they did not feel adequately trained to deliver teaching remotely (Ashworth, Putwain, et al., 2022). Thus, in event of a future lockdown, it is vital that education staff receive increased support to be able to manage the extra workload effectively.

Parents were motivated to help sustain their child’s learning whilst at home but found it more difficult to oversee home-schooling for children of different ages, as well as supervising home-schooling for younger children who required more encouragement. Some parents reported feeling overwhelmed and noted conflict between them and their children over completing schoolwork which is consistent with other studies (Nayir & Sari, 2021; Thorell et al., 2022). This also aligns with research by Schmidt et al. (2021), who found that on home-school days, parents reported a higher amount of negative parent–child interactions, lower child and parental positive affect, and higher child negative affect.

Schools provided a more structured education with live lessons, a heavier workload, and more teacher feedback by the second lockdown. Less parent input was required, and parents viewed these changes positively. However, for some adolescents, the increased workload caused anxiety and psychological distress (Ashworth, Putwain, et al., 2022; Elharake et al., 2022; Irwin et al., 2022; Liang et al., 2020; Rajmil et al., 2021; Thorell et al., 2022). Planning and routine were essential to the maintenance of education and having schoolwork to do helped prevent boredom at home.

In most households, at least one parent worked from home. This study suggests that it was crucial to have adequate technology and designated workspaces for parents and adolescents. This aligns with research showing that inadequate space at home is linked to elevated stress in parents and poorer outcomes of home-schooling (Aznar et al., 2021; Child Poverty Action Group, 2020). As access to technology disproportionately negatively impacts families from low-income backgrounds (Hernandez & Roberts, 2018), it is important to recognise the value of the provision of appropriate technology, particularly for pupils who are eligible for free school meals (FSM). While there are 2.7 million pupils in the UK receive FSM (DfE, 2021), only 200,000 devices were provided by the Government at the beginning of the first lockdown (DfE, 2021). Thus, it is of upmost importance for the Government to ensure that all pupils eligible for FSM have the relevant technology to enable them to engage in schoolwork from home.

Parents enjoyed working from home, found it more productive, and helped them balance work with childcare. Difficulties included less interaction with colleagues, difficulty learning new skills from home, maintaining contact with friends, tiredness, and balancing caring responsibilities. The responsibility of overseeing their child’s schoolwork whilst working was challenging for parents, which aligns with previous research (e.g. Briggs, 2020; Parczewska, 2021), and keyworker parents felt guilty about putting their families at possible risk. Several households carried on as usual during lockdowns and felt grateful for the resources such as space and technology that enabled them to do this.

However, parents felt that their adolescents in particular missed out on life experiences due to the pandemic. For example, exams were cancelled, no transition from primary to secondary school occurred, adolescents had fewer opportunities to socialise and make friends, participated in less physical activity, and could not try new afterschool clubs and activities. This impact may be particularly prominent given the age of the adolescents in the current study (11–13 years). Early adolescence is a time when adolescents are going through key stages of transition, forming new peer relations, and are exploring their new school environment (Jindal-Snape et al., 2020). It is also a developmental period characterised by increased autonomy, and interaction with peers is of great importance (Van Ryzin et al., 2009). Furthermore, not being able to see family members (such as parents and grandparents) had a negative impact on parents and adolescents during periods of lockdown. Parents also reported an increased need for them to provide care to relatives. Some parents and adolescents also experienced a decline in their physical and mental health. Similar findings have been reported regarding the impact on parents and adolescents’ mental health (Ashworth, Putwain, et al., 2022; Elharake et al., 2022; Irwin et al., 2022; Liang et al., 2020; Rajmil et al., 2021; Thorell et al., 2022).

To manage during lockdowns and maintain well-being, parents and adolescents engaged in self-care and coping strategies such as exercise, maintaining normal activities, and having fun. Our findings offer insight into the adaptation and resilience occurring among early adolescents at this time, and demonstrate the active role they took in caring for their well-being. Their engagement in personal development and self-care reflects positive youth development, positioning adolescents as agents of their own development and growth (Larson, 2006). It has previously been argued that education systems are at odds with this need for self-determination in adolescence, with little scope for adolescents to exercise autonomy (Eccles et al., 1993). Thus, the COVID-19 pandemic may represent an opportunity to reflect on the structure of our education system and the demands placed on our adolescents.

While adolescents often rely on social support as a coping strategy, this is typically sought through peers (Cicognani, 2011; Gelhaar et al., 2007). However, our findings indicate that during lockdown, adolescents spent more time engaging in activities as a family, playing together with siblings and/or parents, and connecting with family virtually. . The increased family bonding may have also been a factor that helped adolescents cope during the pandemic. This is mirrored in quantitative findings, where evidence has shown that strong family connection was associated with lower externalising difficulties over the pandemic in this age group (Ashworth, Putwain, et al., 2022).

### Strengths and limitations of the study

To our knowledge this is the first dyad study to include qualitative data from both adolescents and their parents/carers. A strength of the dyad study was that we spoke to both adolescents and their parents to get multiple perspectives on the topic at different stages of the lockdown measures. Dyadic analysis in qualitative research allows for researchers to better understand and identify overlaps and contrasts between two groups of people interviewed, particularly if they have been interviewed separately. For this study, this was a pivotal stage in bringing together parents’ and adolescents’ experiences in a transparent and clear way, creating one overall dyadic summary. Dyadic analysis allowed for researchers to better understand and identify overlaps and contrasts between two groups of people interviewed, particularly if they were interviewed separately. Drawing upon such dyadic coping models enhanced our understanding of why parents and adolescents may have engaged in certain behaviours in the context of how each was impacted by the COVID-19 restrictions, as well as providing insights into how families who may find themselves in similar situations in the future can be better supported. The process of dyadic analysis has previously been shown to provide a deeper understanding of two relational groups of interview data (Eisikovits & Koren, 2010; White & Newman, 2016).

There were limitations to this study. The sample was not representative and two of the schools the adolescents attended were academically selective and one fee paying. Pupils attending academically selective schools may have more available resources than pupils attending schools in more deprived areas. More deprived households may not have the same access to wifi, technology and a garden. There have been fears that home-schooling could amplify the attainment disparity between children from deprived backgrounds and those from more affluent households (Aznar et al., 2021; Bubb & Jones, 2020; Child Poverty Action Group, 2020). Previous studies have found that children from the poorest families are less likely to have sufficient technology (Cullinane & Montacute, 2020) and have fewer resources to support home learning, such as a parent/carer who can help (Guterman & Neuman, 2018). Another limitation was that interviews were conducted remotely. Building rapport during a remote interview may be more challenging than a face-to-face interview. Furthermore, some participants may feel uncomfortable talking about sensitive issues over videocall (Deakin & Wakefield, 2014; Seitz, 2016). O’Sullivan et al. (2020) also noted that it is harder to read non-verbal communication and silences during an online interview. However, other research has suggested that online interviews may have potential benefits such as being less intrusive, more convenient, and the participant may feel safer and more comfortable (Dodds & Hess, 2021). Another limitation is the time period between the two interviews conducted with children and then their parents. Specifically, the COVID-19 severity changes day by day might have led to the participants having different experiences and feelings across time. This may have confounded the present study’s findings. A final limitation is that the parents and adolescents volunteered to be interviewed. Different themes may have been identified from a broader sample.

### Conclusion

In conclusion, when advising others going through the same situation, parents and adolescents suggested being realistic about the situation, helping and supporting each other, putting mental health and happiness first, not comparing self to others, keeping entertained, following guidelines/keeping safe, enjoying time together, exercise/physical activities, playing together, virtual reality, talking, and taking a break from work. Our findings will help to inform policy and practice for supporting adolescents and parents in the future, including: consistency in schools communication with both parents and adolescents when home-schooling; guidance for families on a healthy work–life balance if the need for home-schooling arises in the future; providing additional recreational activities for adolescents to help them gain social skills that they may have been able to develop during the COVID-19 pandemic; the promotion of positive coping strategies; and, the provision of technology and resources for adolescents, schools and families to reduce inequalities.

*Author contribution*: Substantial contributions to the conception or design of the work: PS, EA; data collection: AH, JK; interpretation of the data: PS, EA, AH, JK, JC; drafting the work: PS, EA, AH, JK, JC; revising the work critically for important intellectual content: PS, EA, AH, JK, JC; final approval of the version to be published: PS, EA, AH, JK, JC. The corresponding author attests that all listed authors meet authorship criteria and that no others meeting the criteria have been omitted. All authors have read and agreed to the published version of the manuscript.

*Compliance with ethical standards*: All procedures performed in studies involving human participants were in accordance with the ethical standards of the institutional and/or national research committee and with the 1964 Helsinki Declaration and its later amendments or comparable ethical standards. The study was approved by the Liverpool John Moores University Research Ethics Committee (20/NSP/037).

*Consent to participate*: Informed consent was obtained from all individual participants included in the study. Informed consent was also obtained from parents/legal guardians for the adolescents interviewed.

## Data Availability

The datasets used and/or analysed during the current study are available from the corresponding author on reasonable request.

## References

[CIT0001] Allen, R. E., & Wiles, J. L. (2016). A rose by any other name: Participants choosing research pseudonyms. *Qualitative Research in Psychology*, *13*(2), 149–165. 10.1080/14780887.2015.1133746

[CIT0002] Arksey, H., & Knight, P. T. (1999). *Interviewing for social scientists: An introductory resource with examples*. Sage.

[CIT0003] Ashworth, E., Hunt, A., Chopra, J., Eames, C., Putwain, D. W., Duffy, K., Kirkby, J., McLoughlin, S., & Saini, P. (2022). Adolescents’ lockdown-induced coping experiences (ALICE) study: A qualitative exploration of early adolescents’ experiences of lockdown and reintegration. *The Journal of Early Adolescence*, *42*(4), 514–541. 10.1177/02724316211052088

[CIT0004] Ashworth, E., Putwain, D. W., McLoughlin, S., Saini, P., Chopra, J., Rosser, B., & Eames, C. (2022). Ordinary magic in extraordinary circumstances: Factors associated with positive mental health outcomes for early adolescents during the COVID-19 pandemic. *Adversity and Resilience Science*, *3*(1), 65–79. 10.1007/s42844-022-00054-0 35128460 PMC8801386

[CIT0005] Aznar, A., Sowden, P., Bayless, S., Ross, K., Warhurst, A., & Pachi, D. (2021). Home-schooling during COVID-19 lockdown: Effects of coping style, home space, and everyday creativity on stress and home-schooling outcomes. *Couple and Family Psychology: Research and Practice*, *10*(4), 294–312. 10.1037/cfp0000182

[CIT0006] Branquinho, C., Kelly, C., Arevalo, L. C., Santos, A., & Gaspar de Matos, M. (2020). “Hey, we also have something to say”: A qualitative study of Portuguese adolescents’ and young people’s experiences under COVID-19. *Journal of Community Psychology*, *48*(8), 2740–2752. 10.1002/jcop.22453 33001455 PMC7537124

[CIT0007] Braun, V., & Clarke, V. (2006). Using thematic analysis in psychology. *Qualitative Research in Psychology*, *3*(2), 77–101. 10.1191/1478088706qp063oa

[CIT0008] Braun, V., & Clarke, V. (2019). Reflecting on reflexive thematic analysis. *Qualitative Research in Sport, Exercise and Health*, *11*(4), 589–597. 10.1080/2159676X.2019.1628806

[CIT0009] Briggs, D. C. (2020). COVID-19: The effect of lockdown on children’s remote learning experience – Parents’ perspective. *Journal of Education, Society and Behavioural Science*, *33*, 42–52. 10.9734/jesbs/2020/v33i930257

[CIT0010] British Medical Association. (2020). *The impact of COVID-19 on mental health in England; Supporting services to go beyond parity of esteem*. https://www.bma.org.uk/media/2774/emb07072020-bma-mental-health-paper.pdf

[CIT0011] Bubb, S., & Jones, M.-A. (2020). Learning from the COVID-19 home-schooling experience: Listening to pupils, parents/carers and teachers. *Improving Schools*, *23*(3), 209–222. 10.1177/1365480220958797

[CIT0012] Calear, A. L., McCallum, S., Morse, A. R., Banfield, M., Gulliver, A., Cherbuin, N., Farrer L. M., Murray, K., Rodney, Harris, R. M., & Batterham, P. J. (2022). Psychosocial impacts of home-schooling on parents and caregivers during the COVID-19 pandemic. *BMC Public Health*, *22*(1), 119. 10.1186/s12889-022-12532-2 35039044 PMC8763398

[CIT0013] Cheng, Z., Mendolia, S., Paloyo, A., Savage, D., & Tani, M. (2021). Working parents, financial insecurity, and childcare: Mental health in the time of COVID-19 in the UK. *Review of Economics of the Household*, *19*(1), 123–144. 10.1007/s11150-020-09538-3 33456425 PMC7802611

[CIT0014] Child Poverty Action Group. (2020). *The cost of learning in lockdown*. https://cpag.org.uk/policy-and-campaigns/report/cost-learning-lockdown-family-experiences-school-closures

[CIT0015] Child Poverty Action Group. (2021). *Digital exclusion during the pandemic*. https://cpag.org.uk/policy-and-campaigns/briefing/digital-exclusion-during-pandemic

[CIT0016] Cicognani, E. (2011). Coping strategies with minor stressors in adolescence: Relationships with social support, self-efficacy, and psychological well-being. *Journal of Applied Social Psychology*, *41*(3), 559–578. 10.1111/j.1559-1816.2011.00726.x

[CIT0017] Cullinane, C., & Montacute, R. (2020). *Research brief: April 2020: COVID-19 and social mobility impact brief# 1: School shutdown*. https://dera.ioe.ac.uk/35356/1/COVID-19-Impact-Brief-School-Shutdown.pdf

[CIT0018] Deakin, H., & Wakefield, K. (2014). Skype interviewing: Reflections of two PhD researchers. *Qualitative Research*, *14*(5), 603–616. 10.1177/1468794113488126

[CIT0501] Department for Education. (2021). Schools, pupils and their characteristics: Academic year 2019/20.

[CIT0019] Dodds, S., & Hess, A. C. (2021). Adapting research methodology during COVID-19: Lessons for transformative service research. *Journal of Service Management*, *32*(2), 203–217. 10.1108/JOSM-05-2020-0153

[CIT0020] Dvorsky, M. R., Breaux, R., & Becker, S. P. (2021). Finding ordinary magic in extraordinary times: Child and adolescent resilience during the COVID-19 pandemic. *European Child & Adolescent Psychiatry*, *30*(11), 1829–1831. 10.1007/s00787-020-01583-8 32613259 PMC7327857

[CIT0021] Eccles, J. S., Midgley, C., Wigfield, A., Buchanan, C. M., Reuman, D., Flanagan, C., & Mac Iver, D. (1993). Development during adolescence: The impact of stage-environment fit on young adolescents' experiences in schools and in families. *American Psychologist*, *48*(2), 90–101. 10.1037/0003-066X.48.2.90 8442578

[CIT0022] Eder, D., & Fingerson, L. (2003). Interviewing children and adolescents. In James A. Holstein & Jaber F. Gubrium (Eds.), *Inside interviewing: New lenses, new concerns* (pp. 33–53). SAGE Publications.

[CIT0023] Eisikovits, Z., & Koren, C. (2010). Approaches to and outcomes of dyadic interview analysis. *Qualitative Health Research*, *20*(12), 1642–1655. 10.1177/1049732310376520 20663940

[CIT0024] Elharake, J. A., Akbar, F., Malik, A. A., Gilliam, W., & Omer, S. B. (2022). Mental health impact of COVID-19 among children and college students: A systematic review. *Child Psychiatry & Human Development*. 10.1007/s10578-021-01297-1 PMC8747859 35013847

[CIT0025] Galletta, A. (2013). *Mastering the semi-structured interview and beyond: From research design to analysis and publication* (Vol. 18). NYU Press.

[CIT0026] Gelhaar, T., Seiffge-Krenke, I., Borge, A., Cicognani, E., Cunha, M., Loncaric, D., Macek, P., Steinhausen, H.-C., & Metzke, C. (2007). Adolescent coping with everyday stressors: A seven-nation study of youth from central, eastern, southern, and northern Europe. *European Journal of Developmental Psychology*, *4*(2), 129–156. 10.1080/17405620600831564

[CIT0027] Gov.UK. (2021). *Coronavirus (COVID 19)*. https://www.gov.uk/coronavirus

[CIT0028] Guterman, O., & Neuman, A. (2018). Personality, socio-economic status and education: Factors that contribute to the degree of structure in home-schooling. *Social Psychology of Education*, *21*(1), 75–90. 10.1007/s11218-017-9406-x

[CIT0029] Hernandez, K., & Roberts, T. (2018). *Leaving no-one behind in a digital world*. Digital and Technology Cluster Institute of Development Studies. https://assets.publishing.service.gov.uk/media/5c178371ed915d0b8a31a404/Emerging_Issues_LNOBDW_final.pdf

[CIT0030] Horesh, D., & Brown, A. D. (2020). Traumatic stress in the age of COVID-19: A call to close critical gaps and adapt to new realities. *Psychological Trauma: Theory, Research, Practice, and Policy*, *12*(4), 331–335. 10.1037/tra0000592 32271070

[CIT0031] Irwin, M., Lazarevic, B., Soled, D., & Adesman, A. (2022). The COVID-19 pandemic and its potential enduring impact on children. *Current Opinion in Pediatrics*, *34*(1), 107–115. 10.1097/MOP.0000000000001097 34923563 PMC8728751

[CIT0032] Jindal-Snape, D., Hannah, E. F. S., Cantali, D., Barlow, W., & MacGillivray, S. (2020). Systematic literature review of primary–secondary transitions. *Review of Education*, *8*(2), 526–566. 10.1002/rev3.3197

[CIT0033] Kerr, M., Rasmussen, H., Fanning, K., & Braaten, S. (2021). Parenting during COVID-19: A study of parents’ experiences across gender and income levels. *Family Relations*, *70*(5), 1327–1342. 10.1111/fare.12571 34548726 PMC8444790

[CIT0034] Larson, R. (2006). Positive youth development, willful adolescents, and mentoring. *Journal of Community Psychology*, *34*(6), 677–689. 10.1002/jcop.20123

[CIT0035] Lee, J. (2020). Mental health effects of school closures during COVID-19. *The Lancet Child & Adolescent Health*, *4*(6), 421. 10.1016/S2352-4642(20)30109-7 32302537 PMC7156240

[CIT0036] Liang, L., Ren, H., Cao, R., Hu, Y., Qin, Z., Li, C., & Mei, S. (2020). The effect of COVID-19 on youth mental health. *Psychiatric Quarterly*, *91*(3), 841–852. 10.1007/s11126-020-09744-3 32319041 PMC7173777

[CIT0037] Long, R., & Danechi, S. (2021). *Teacher recruitment and retention in England*. House of Commons Library*.* https://researchbriefings.files.parliament.uk/documents/CBP-7222/CBP-7222.pdf

[CIT0038] Manning, J., & Kunkel, A. (2015). Qualitative approaches to dyadic data analyses in family communication research: An invited essay. *Journal of Family Communication*, *15*(3), 185–192. 10.1080/15267431.2015.1043434

[CIT0039] Millar, R., Quinn, N., Cameron, J., & Colson, A. (2020). *Impacts of lockdown on the mental health and wellbeing of children and adolescents. Considering evidence within the context of the individual, the family, and education*. Mental Health Foundation.

[CIT0040] Nayir, F., & Sari, T. (2021). Identifying parents’ home-schooling experience during COVID-19 period. *Asian Journal of Distance Education*, *16*, 156–170. 10.5281/zenodo.4888516

[CIT0041] O’Sullivan, K., Clark, S., McGrane, A., Rock, N., Burke, L., Boyle, N., Joksimovic, N., & Marshall, K. A. (2021). A qualitative study of child and adolescent mental health during the COVID-19 pandemic in Ireland. *International Journal of Environmental Research and Public Health*, *18*(3), 1062. 10.3390/ijerph18031062 33504101 PMC7908364

[CIT0042] O’Sullivan, K., McGrane, A., Clark, S., & Marshall, K. (2020). Exploring the impact of home-schooling on the psychological wellbeing of Irish families during the novel coronavirus (COVID-19) pandemic: A qualitative study protocol. *International Journal of Qualitative Methods*, *19*. 10.1177/1609406920980954

[CIT0043] Parczewska, T. (2021). Difficult situations and ways of coping with them in the experiences of parents homeschooling their children during the COVID-19 pandemic in Poland. *Education 3-13*, *49*(7), 889–900. 10.1080/03004279.2020.1812689

[CIT0044] Rajmil, L., Hjern, A., Boran, P., Gunnlaugsson, G., de Camargo, O. K., & Raman, S. (2021). Impact of lockdown and school closure on children’s health and well-being during the first wave of COVID-19: A narrative review. *BMJ Paediatrics Open*, *5*(1), e001043. 10.1136/bmjpo-2021-001043 34192198 PMC8154298

[CIT0045] Schmidt, A., Kramer, A. C., Brose, A., Schmiedek, F., & Neubauer, A. B. (2021). Distance learning, parent–child interactions, and affective wellbeing of parents and children during the COVID-19 pandemic: A daily diary study. *Developmental Psychology*, *57*(10), 1719–1734. 10.1037/dev0001232 34807692

[CIT0046] Seitz, S. (2016). Pixilated partnerships, overcoming obstacles in qualitative interviews via Skype: A research note. *Qualitative Research*, *16*(2), 229–235. 10.1177/1468794115577011

[CIT0047] Sevilla, A., & Smith, S. (2020). *Baby steps: The gender division of childcare during the COVID-19 pandemic* (IZA Discussion Papers 13302). Institute of Labor Economics. 10.1093/oxrep/graa027 PMC7499756 40504206

[CIT0048] Solmi, M., Estradé, A., Thompson, T., Agorastos, A., Radua, J., Cortese, S., Dragioti, E., Leisch, F., Vancampfort, D., Thygesen, L. C., Aschauer, H., Schloegelhofer, M., Akimova, E., Schneeberger, A., Huber, C. G., Hasler, G., Conus, P., Cuénod, K. Q. D., von Känel, R., … Correll, C. U. (2022). Physical and mental health impact of COVID-19 on children, adolescents, and their families: The collaborative outcomes study on health and functioning during infection times – Children and adolescents (COH-FIT-C&A). *Journal of Affective Disorders*, *299*, 367–376. 10.1016/j.jad.2021.09.090 34606810 PMC8486586

[CIT0049] Thorell, L., Skoglund, C., Giménez, A., Baeyens, D., Fuermaier, A., Groom, M., Mammarella, I. C., van der Oord, S., van den Hoofdakker, B. J., Luman, M., de Miranda, D. M., Siu, A. F. Y., Steinmayr, R., Idrees, I., Soares, L. S., Sörlin, M., Luque, J. L., Moscardino, U. M., Roch, M., … Christiansen, H. (2022). Parental experiences of homeschooling during the COVID-19 pandemic: Differences between seven European countries and between children with and without mental health conditions. *European Child & Adolescent Psychiatry*, *31*(4), 649–661. 10.1007/s00787-020-01706-1 33415470 PMC7790054

[CIT0050] Van Ryzin, M. J., Gravely, A. A., & Roseth, C. J. (2009). Autonomy, belongingness, and engagement in school as contributors to adolescent psychological well-being. *Journal of Youth and Adolescence*, *38*(1), 1–12. 10.1007/s10964-007-9257-4 19636787

[CIT0051] White, N., & Newman, E. (2016). Shared recovery: Couples’ experiences after treatment for colorectal cancer. *European Journal of Oncology Nursing*, *21*, 223–231. 10.1016/j.ejon.2015.10.008 26643399

[CIT0052] Yardley, L. (2000). Dilemmas in qualitative health research. *Psychology & Health*, *15*(2), 215–228. 10.1080/08870440008400302

[CIT0053] Yerkes, M. A., André, S. C. H., Besamusca, J. W., Kruyen, P. M., Remery, C. L. H. S., van der Zwan, R., Beckers, D. G. J., & Geurts, S. A. E. (2020). ‘Intelligent’ lockdown, intelligent effects? Results from a survey on gender (in)equality in paid work, the division of childcare and household work, and quality of life among parents in the Netherlands during the Covid-19 lockdown. *PLoS One*, *15*(11), e0242249. 10.1371/journal.pone.0242249 33253238 PMC7703961

